# Comparative Efficacy and Safety of Thrombopoietin Receptor Agonists in Adults With Thrombocytopenia: A Systematic Review and Network Meta-analysis of Randomized Controlled Trial

**DOI:** 10.3389/fphar.2021.704093

**Published:** 2021-07-28

**Authors:** Junzhu Deng, Haiyang Hu, Feihong Huang, Chunlan Huang, Qianqian Huang, Long Wang, Anguo Wu, Jing Yang, Dalian Qin, Wenjun Zou, Jianming Wu

**Affiliations:** ^1^ Department of Chinese Materia Medica, School of Pharmacy, Chengdu University of Traditional Chinese Medicine, Chengdu, China; ^2^ Laboratory of Chinese Materia Medica, Department of Pharmacology, School of Pharmacy, Southwest Medical University, Luzhou, China; ^3^ Institute of Cardiovascular Research, the Key Laboratory of Medical Electrophysiology, Ministry of Education of China, Medical Key Laboratory for Drug Discovery and Druggability Evaluation of Sichuan Province, Luzhou Key Laboratory of Activity Screening and Druggability Evaluation for Chinese Materia Medica, Luzhou, China; ^4^ Stem Cell Laboratory and Department of Hematology, Affiliated Hospital of Southwest Medical University, Luzhou, China; ^5^ Department of Pharmacy, Affiliated Hospital of Southwest Medical University, Luzhou, China

**Keywords:** thrombopoietin receptor agonist, thrombocytopenia, platelet, platelet response, network meta-analysis

## Abstract

Thrombopoietin receptor agonists (TPO-RAs) play a crucial role in stimulating thrombopoiesis. However, conventional meta-analyses have shown inconsistent results regarding the efficacy of thrombopoietin receptor agonists versus placebo. Therefore, we performed a network meta-analysis to assess the effects of five TPO-RAs *via* indirect comparison. For this network meta-analysis, we considered randomized trials that included any of the following interventions: avatrombopag, lusutrombopag, eltrombopag, romiplostim, recombinant human thrombopoietin (rhTPO). We searched the *Medline*, *PubMed*, *Embase*, *the Cochrane Library*, and *Web of Science* databases for randomized controlled clinical trials from inception to January 31, 2021. We use randomized controlled clinical trials of TPO-RAs for treatment of immune thrombocytopenia in adults. The primary outcome was the number of patients achieving platelet response which was defined as the achievement of a platelet count of more than 30 or 50 cells × 10^9^/L in the absence of rescue therapy, and the secondary outcome was the therapy-related serious adverse events and incidence of bleeding episodes. To obtain the estimates of efficacy and safety outcomes, we performed a random-effects network meta-analysis. These estimates were presented as odds ratios with 95% confidence intervals. We use surface under the cumulative ranking probabilities to rank the comparative effects and safety of all drugs against the placebo. In total, 2,207 patients were analyzed in 20 clinical trials. All preparations improved the point estimates of platelet response when compared with the placebo. Avatrombopag and lusutrombopag had the best platelet response compared to the placebo, the former had a non-significant advantage compared to the latter [odds ratio (OR) = 1.91 (95% confidence interval: 0.52, 7.05)]. The treatments were better than eltrombopag, romiplostim, rituximab, and rhTPO + rituximab, with corresponding ORs of 3.10 (1.01, 9.51), 9.96 (2.29, 43.29), 33.09 (8.76, 125.02), and 21.31 (3.78, 119.98) for avatrombopag and 1.62 (0.63, 4.17), 5.21 (1.54, 17.62), 17.34 (5.15, 58.36), and 11.16 (2.16, 57.62) for lusutrombopag. Regarding bleeding, the placebo group had the highest probability of bleeding, whereas lusutrombopag had the lowest risk of bleeding when compared to the placebo. Adverse events were slightly higher in patients receiving rituximab than in those receiving placebo or other treatments. Overall, this meta-analysis showed that avatrombopag may yield the highest efficacy because it has the most favorable balance of benefits and acceptability.

## Introduction

Immune thrombocytopenia (ITP) is characterized by a decrease in the number of platelets in peripheral blood. The definition of ITP is a platelet count below 100 × 10^9^/L and clinical signs of bleeding (Wolfromm and Dallemagne, 2018). If platelet counts are between 50 × 10^9^/L and 100 × 10^9^/L, we consider to mild thrombocytopenia that usually does not lead to clinical symptoms. However, persistent platelet counts below 30 × 10^9^/L may be associated with spontaneous bruising and death (Izak and Bussel, 2014). ITP is a clinically common hemorrhagic disease characterized by a decrease in the number of platelets in the peripheral blood with an incidence around 5.1 to 5.5/100,000 person-year (Lee et al., 2017). Because of its difficulty in curing and potential bleeding risk, it seriously affects people’s quality of life. ITP can be divided into primary and secondary. Primary ITP is an autoimmune disorder occurring in response to an unknown stimulus, occurring due to the loss of immune tolerance to platelet autoantigens in patients. Secondary ITP is triggered by many factors including autoimmune diseases, viral infections, human immunodeficiency virus and certain drugs (Neunert et al., 2011). Under immune-mediated processes, excessive destruction of platelets and inhibition of platelet generation result in decreased platelet counts.

There are several options for treating thrombocytopenia, with corticosteroids and splenectomy as the initial treatments. Corticosteroids are the first-line treatment of adults, typically with a prednisone regimen of 1 mg/kg/day. However, relapse and adverse events are common in patients with corticosteroids (Cuker et al., 2016). If corticosteroids do not induce a response, splenectomy is considered the second-line treatment. The risk of postoperative infection, thrombosis, and other complications after splenectomy is rather high (Lambert and Gernsheimer, 2017). So splenectomy has been rarely used in clinics. Recently, many immunosuppressants and combination regimens have been used (second-line), but their long-term response rates are unsatisfactory (Lambert and Gernsheimer, 2017). In recent years, thrombopoietin receptor agonists (TPO-RAs) have been actively used to stimulate platelet production and reduce the risk of bleeding. Many randomized double-blind placebo-controlled trials have been carried out to prove its effectiveness. A recent meta-analysis of romiplostim and eltrombopag has demonstrated that TPO-RAs greatly increased the number of platelets and reduced the bleeding events (Zhang et al., 2017). Another meta-analysis of TPO-RAs and rituximab illustrated the efficacy and safety of TPO-RAs are better than rituximab (Yang et al., 2019). But there is not a network meta-analysis to assess the effects of five TPO-RAs via indirect comparison. At present, there are five kinds of TPO-RAs, including avatrombopag, lusutrombopag, eltrombopag, romiplostim, and rhTPO. Avatrombopag is a small molecule TPO-RA that mimics the biological effects of endogenous TPO on platelet production. Doptelet^®^ (avatrombopag tablet) was approved by the US Food and Drug Administration (FDA) on May 21, 2018, for treating other thrombocytopenic disorders including ITP and chronic liver disease-induced thrombocytopenia (Shirley, 2018). As a chemically synthesized and orally active small-molecule TPO-RA, lusutrombopag can activate the signal transduction pathway in the same manner as endogenous TPO, thereby upregulating platelet production (Neunert et al., 2011). Lusutrombopag was approved in Japan in 2015 for use in patients with thrombocytopenia and chronic liver disease who are undergoing invasive procedures (Kim, 2016). Eltrombopag is an oral, small molecule, non-peptide TPO-RA. By interacting with the transmembrane domain of the receptor, this drug initiates thrombopoietin-receptor signaling, thereby inducing cell proliferation, differentiation and maturation in the megakaryocytic lineage (Sellers et al., 2004). Romiplostim is a novel peptide molecule that stimulates the megakaryocytopoiesis and increases the platelet count in the same manner as TPO (Wang et al., 2004). As a full-length and glycosylated TPO developed by Shenyang Sunshine Pharmaceutical Co., Ltd., rhTPO was approved by the China State Food and Drug Administration as a second-line option for ITP (Zhou et al., 2015). The course of treatment of TPO-RAs is uncertain. The overall goal of the duration is to achieve platelet counts ≥50 × 10^9^ (Jurczak et al., 2018).

To date, there has been no comprehensive analysis of the efficacy and safety of five TPO-RAs in patients with ITP. Conventional meta-analysis can only compare the therapeutic effects of a TPO-RA versus placebo directly from head to head. The therapeutic superiority of each regimen cannot be determined through simple comparison of outcomes in different studies or conventional meta-analysis. Network meta-analysis (NMA) is the synthesis of information to assess the comparative effectiveness of more than two alternative treatment options (Cipriani et al., 2013). It allows integrated analysis of all randomized controlled trials (RCTs) comparing different TPO-RAs head to head or with placebo while fully respecting randomization. Therefore, in this study, we assessed the effectiveness of different TPO-RAs for ITP in increasing the platelet count by integrating all the available direct and indirect evidence through network meta-analysis. Network of eligible comparisons for the multiple treatments meta-analysis is presented in the form of network evidence map. The width of the lines is proportional to the number of trials comparing each pair of treatments. The dimension of each node is representative of the number of randomly assigned participants (sample size) (Cipriani et al., 2013). Network forest plot plays an important role in the effect of each treatment. Through the pooled effect of each treatment, we can acquire information about the test for the inconsistency model. The surface under the cumulative ranking curve (SUCRA) is used to determine relative rankings of treatments. The larger SUCRA is, the more effective the drug is or the more likely the outcome is to happen (Shim et al., 2017).

## Materials and Methods

### Search Strategy

We conducted a literature search to identify all published RCTs based on the search strategies suggested in the Cochrane Handbook for Systematic Reviews of Interventions. The electronic databases *Medline*, *PubMed*, *Embase*, and the *Cochrane Library* were searched for publications listed between each database’s inception date and January 31, 2021. The search terms and MeSH used were mainly “thrombocytopenia,” “TPO,” “thrombopoietin receptor agonists,” “avatrombopag,” “lusutrombopag,” “romiplostim,” “recombinant human thrombopoietin,” “rhTPO.” More detailed search terms were listed in Supplementary Material 5. The American Society of Hematology and ClinicalTrials.gov were searched for unpublished RCTs. When using database retrieval, we limit the article to clinical trial and explode all trees.

### Study Selection

The study selection was performed as follows: 1) Research design: randomized controlled clinical trials of patients with ITP, comprising any of the following interventions: avatrombopag, lusutrombopag, eltrombopag, romiplostim, recombinant human thrombopoietin, or in combination with other drugs. 2) Patients: male or female patients older than 18 years without thrombosis and cardiovascular disease, with the mean baseline platelet count of patients being less than 50 × 10^9^/L. There are no boundaries between countries and races. 3) Outcome measures: at least one of the following three outcomes: the number of patients who achieved platelet response (platelet counts ≥30 or 50 × 10^9^/L) as originally defined by each study, therapy-related serious adverse events, and incidence of bleeding episodes. Trials were excluded for the following reasons: 1) document type (reviews, meeting summaries, letters, etc.), 2) missing or incomplete information on the trial, and 3) patients with diseases related to the blood system.

### Data Extraction

Two investigators independently assessed all trials for eligibility, extracted data by screening the titles and abstracts, and retrieved the full articles if a decision could not be made. In case of disagreement, consensus was reached through discussion. Data extraction was performed independently by the two reviewers. We extracted the trial design, trial size, details of intervention including dose and treatment duration, and patient characteristics such as mean age, sex, mean platelet count, and total number of splenectomies. In addition, data for pooling was extracted, including the total number of subjects, any bleeding events, composite serious adverse events, odds ratio (OR) with 95% confidence interval (CI), and mean with standard deviation of continuous outcomes.

### Quality Assessment

We considered the following aspects for quality assessment: random sequence generation, allocation concealment, blinding, incomplete outcome data, selective reporting, and other biases. Quality assessment was performed using Review Manager (version 5.3; The Cochrane Collaboration, London, United Kingdom). If the opinions of two authors were different, the contradiction was resolved through discussion with a third person.

### Statistical Analysis

We performed meta-analyses using Stata 13 software (StataCorp, College Station, TX, United States). For continuous variables, we calculated the normalized mean difference (MD), and for dichotomous variables, we calculated the odds ratios (ORs). All results were expressed by 95% CI. I^2^ statistics and chi-square tests were used to assess heterogeneity. We used meta-regression to explore the source of heterogeneity. A characteristic was considered a source of heterogeneity if the I^2^ was decreased following its inclusion in the model. Subgroup analysis was then performed. To rank the treatments for an outcome, we used the surface under the cumulative ranking (SUCRA) probabilities, expressing a percentage of the efficacy or safety of every intervention relative to an imaginary intervention (White et al., 2012). A large SUCRA score was considered to indicate a more effective or safer intervention.

## Results

### Study Selection and Characteristics

**FIGURE 1:**
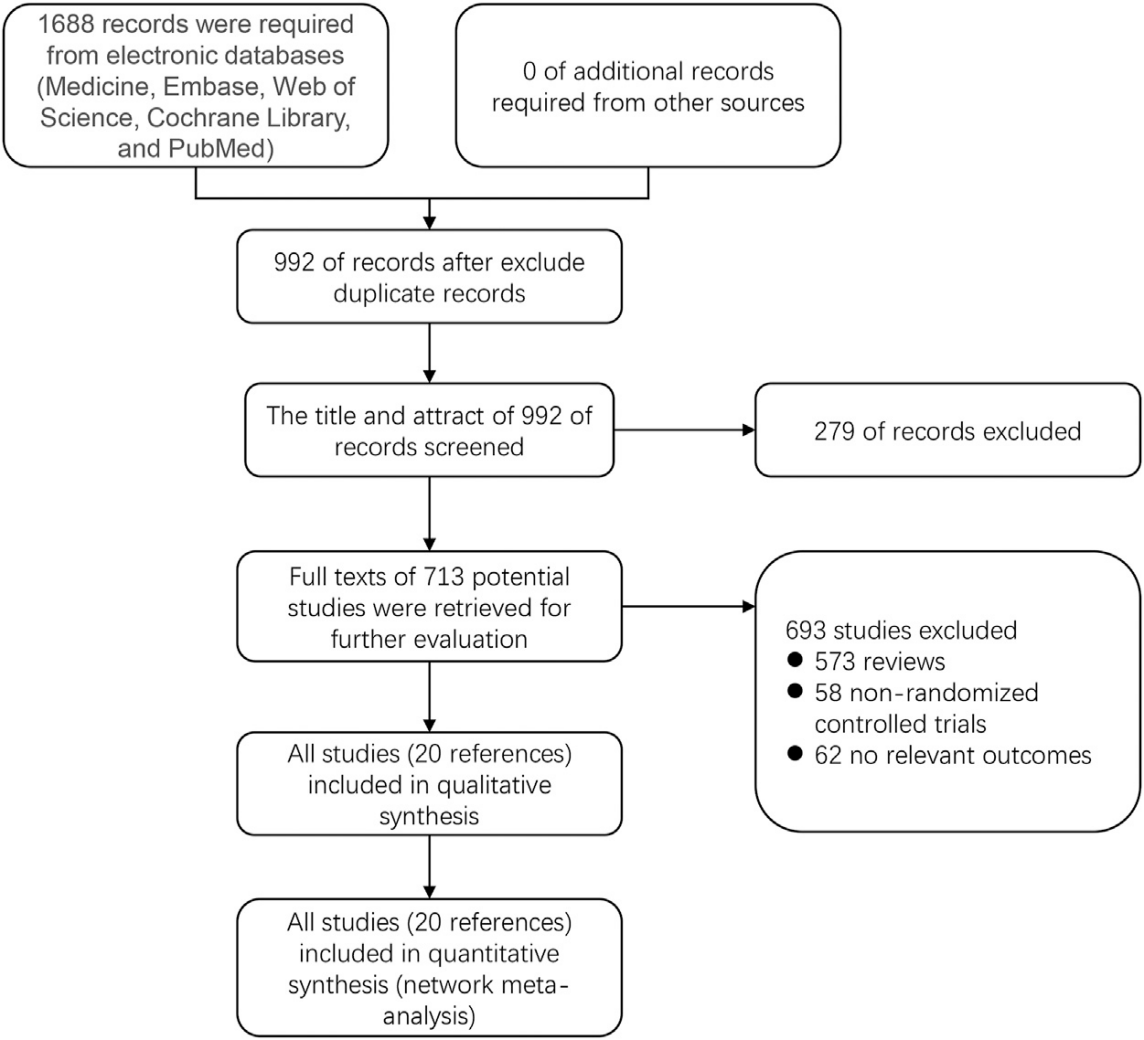
PRISMA flow diagram of the screening and selection process used in the study.

In total, 1,688 articles were identified, of which 696 remained after the duplicates were removed. We excluded 972 reports that did not meet the eligibility criteria. Finally, 20 studies with data for 2,207 participants were available for the network meta-analysis (Figure 1). Because there are no clinical trials about recombinant thrombopoietin versus placebo or other TPO-RAs, we selected rituximab as an intermediate bridge to compare the effects of platelet agonists on platelet count. Six interventional arms were included as follows: four studies with avatrombopag (Bussel et al., 2014; Jurczak et al., 2018; Kuter and Allen, 2018; Terrault et al., 2018), three with lusutrombopag (Hidaka et al., 2019; Peck-Radosavljevic et al., 2019; Tateishi et al., 2019), three with romiplostim (Bussel et al., 2006; Kuter et al., 2008; Shirasugi et al., 2011), seven with eltrombopag (Bussel et al., 2009; Cheng et al., 2011; Afdhal et al., 2012; Tomiyama et al., 2012; Yang et al., 2017; Huang et al., 2018), two with rituximab (RTX) (Arnold et al., 2012; Ghanima et al., 2015), and one with recombinant human thrombopoietin (Zhou et al., 2015). Regarding the control arm, placebo was used in all RCTs except in two studies that selected RTX as the control. The trials were conducted in multiple countries from 2006 to 2019. Approximately two-thirds of the participants (58%) were female. In terms of clinical characteristics, the age of patients ranged from 40–80 years, and the median platelet counts ranged from 9–41×10^9^/L. Table 1 lists the characteristics of the included studies.

**TABLE 1:** Characteristics of randomized controlled trials.

Study ID	Participants	Sex (M/F)	Age (years)	Baseline platelet count (10^9^/L)	Pt N with history of splenectomy, total	Location	Interventions	Duration of treatment
Hisashi Hidaka2019	96 (48/48)	42/57	68.9 ± 6.6/66.8 ± 10.2	40.9 ± 6.3/39.9 ± 6.9	ND	Japan	Lusutrombopag VS placebo	7 days
Ryosuke Tateishi2019	61 (46/15)	26/35	66.8 ± 8.1/70.9 ± 8.6	41.8 ± 13.2/41.8 ± 6.1	ND	Japan	Lusutrombopag VS placebo	7 days
Markus Peck2019	215 (108/107)	81/134	55.2(11.6)/56.1(11.0)	37 0.7 (9.0)/37.4 (7.8)	ND	22 countries	Lusutrombopag VS placebo	7 days
Norah Terrault 2018 (ADAPT -1)	138 (90/48)	97/41	57 ± 29, 78/55 ± 25, 76	31 ± 7/31 ± 7	ND	20 countries	Avatrombopag VS placebo	14 days
Norah Terrault 2018 (ADAPT -2)	113 (70/43)	77/36	62 (20, 86)/58 (27, 77)	33 (±5)/33 (±6)	ND	20 countries	Avatrombopag VS placebo	14 days
Wojciech Jurczak2018	49 (32/17)	18/31	46.4 ± 14.2/41.2 ± 14.7	ND	16	11countries	Avatrombopag VS placebo	6 months
Kuter, David J2018	63 (42/21)	ND	30.6 ± 12.3	ND	ND	United States	Avatrombopag VS placebo	28 days
David J Kuter et al. (2008)	135 (83/52)	54/81	52(21–88)/52(23–88)	16(2–29)/18(2–31	ND	United States, Europe	Romiplostim VS placebo	34 weeks
Yukari Shirasugi et al. (2011)	34(22/12)	10/24	58.5 ± 12.6/47.6 ± 13.4	18.4 ± 8.3/15.8 ± 6	10	Japan	Romiplostim VS placebo	12 weeks
James B. Bussel et al. (2006)	21 (17/4)	6/15	49(19–63)/55(39–64)	16(4–25)/29(6–49	12	United States	Romiplostim VS placebo	6 weeks
Gregory Cheng2011	197(135/62)	61/136	47(34–56)/52.5(43–63)	16(8–22)/16(9–24)	50	23 countries	Eltrombopag VS placebo	6 months
Y T Huang2018	35 (17/18)	6/29	50(24–62)/39.5(22–66)	14(4–27)/13.5(1–26)	ND	CHINA	Eltrombopag VS placebo	6 weeks
Renchi Yang2016	155 (104/51)	38/117	48(18–84)/42(22–66)	14/13.5	25	CHINA	Eltrombopag VS placebo	8 weeks
Nezam H Afdhal2012	292 (145/147)	188/104	52(19–79)/54(19–83)	40(12–62)/40(8–222)	ND	13 countries	Eltrombopag VS placebo	14 days
James B Bussel2009	114 (76/38)	44/70	47(19–84)/51(21–79)	<30	31	23 countries	Eltrombopag VS placebo	6 weeks
Y Tomiyama et al. (2012)	23 (15/8)	9/15	58(26–72)/60.5(38–72)	18.4/15.8	10	23 countries	Eltrombopag VS placebo	6 weeks
James B. Bussel2007	117 (88/29)	44/73	51(18–85)/42(18–85)	15/15	41	23 countries	Eltrombopag VS placebo	6 weeks
Hai Zhou2015	115 (77/38)	40/75	42(13–82)/42.5(12–68)	9(0–30)/12.5(2–30)	12	CHINA	rhTPO + RTX VS RTX	4 weeks
Donald M. Arnold2012	60 (33/27)	25/35	40(30–59)/40(31–59)	15(4–23)/14(10–23)	ND	Canada	RTX VS placebo	4 weeks
Waleed Ghanima2015	110 (64/46)	22/88	46(27–61)/46(28–60)	16(6–27)/21(9–29)	0	Norway, Tunisia, France	RTX VS placebo	4 weeks
Bussel JB2014	64 (59/5)	24/40	53/40	20/15	ND	United States	Avatrombopag VS placebo	28 days

F, female; M, male; NR, not report.

### Risk of Bias in the Included Studies

**FIGURE 2:**
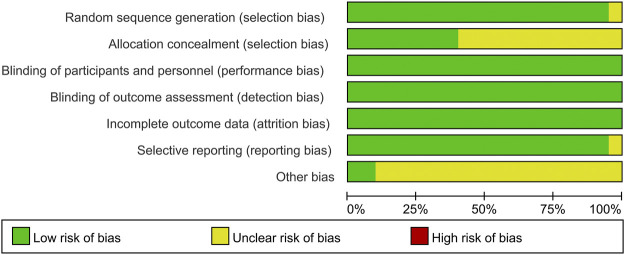
Summary **(A)** and graph **(B)** of the risk of bias in the included trials by Cochrane risk of bias tool. Assessments were based on the reviewers judgment of each domain.

The results of the risk of bias assessment are shown in Figure 2. Generation of random sequences was described in detail for all RCTs, and the method of allocation concealment was described in seven RCTs. Blinding was associated with a low risk of bias. Most RCTs showed a low risk of bias because their protocols and outcomes were well described in each study. Most items were assessed as unclear sources of bias because of insufficient information.

### Outcomes

#### Platelet Response

**FIGURE 3:**
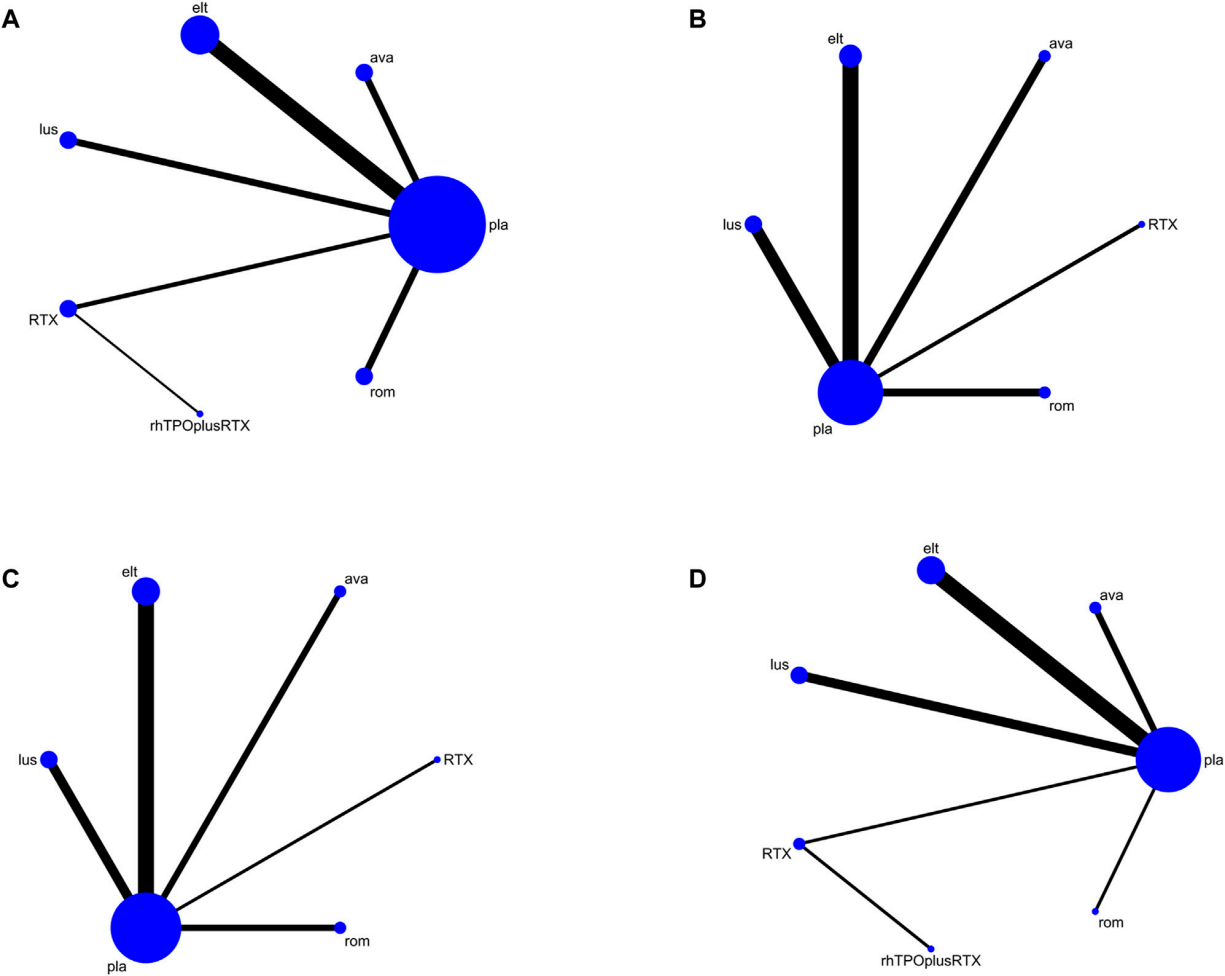
Network map for all outcomes. **(A)** Platelet response. **(B)** Any bleeding. **(C)** Composite serious adverse events. **(D)** Thrombosis (ava, avatrombopag; elt, eltrombopag; rom, romiplostim; RTX, rituximab; lus, lusutrombopag).

**FIGURE 4:**
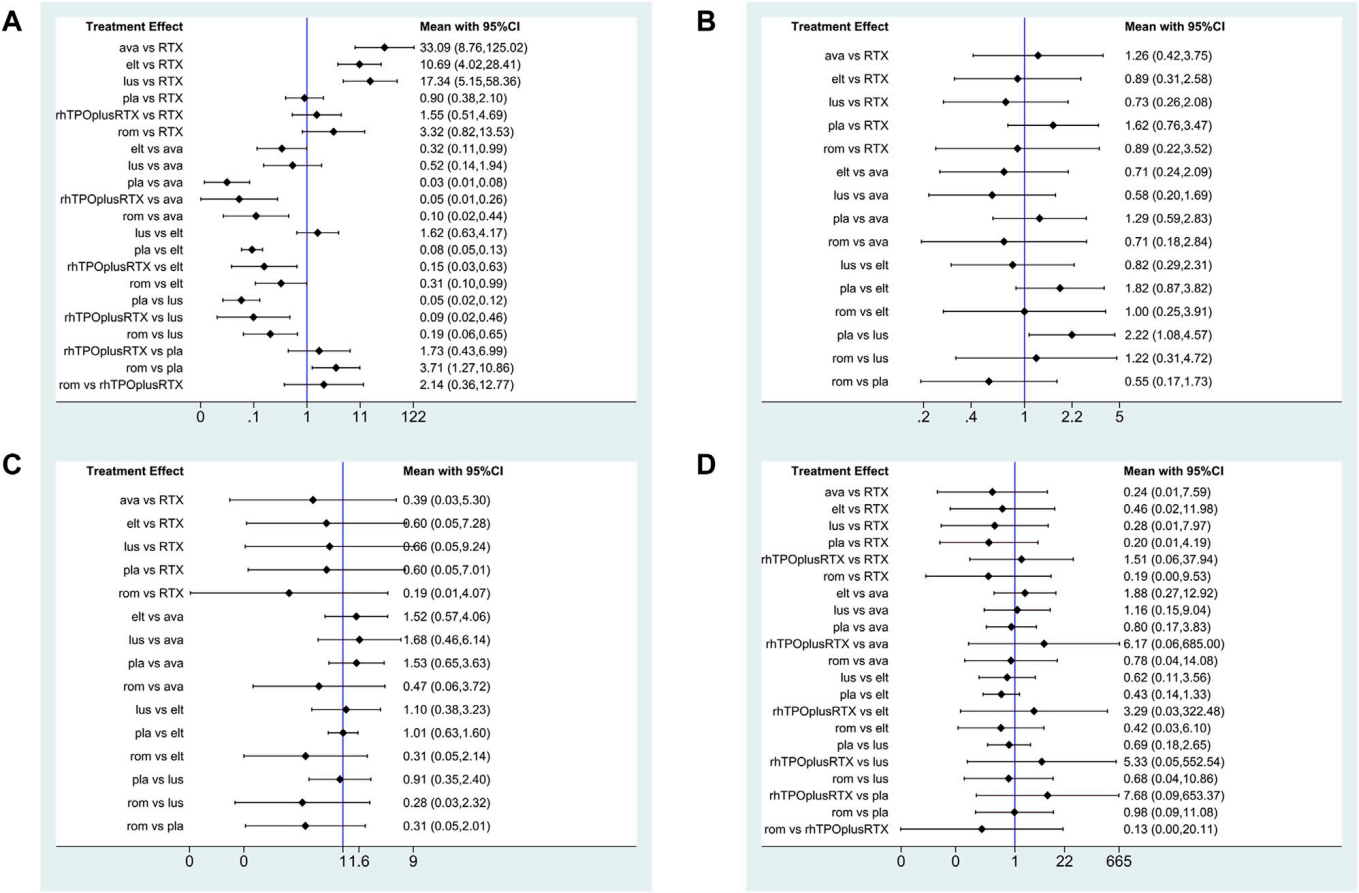
Forest plot of the studies included. **(A)** Platelet response. **(B)** Any bleeding. **(C)** Composite serious adverse events. **(D)** Thrombosis (ava, avatrombopag; elt, eltrombopag; rom, romiplostim; RTX, rituximab; lus, lusutrombopag).

Platelet response (platelet counts ≥30 or 50×10^9^/L) during therapeutic or observational period was regarded as a dichotomous outcome. PR was defined as the proportion of patients who achieved a platelet count of 30 or 50 × 10^9^/L as originally defined by each study, at least once during 4–8 days. Nineteen studies reported platelet response as an outcome. As many as seven treatment arms were included in this analysis (Figure 3A), and the analysis comprised five direct comparisons among six treatments. For all relative treatment comparisons, avatrombopag, lusutrombopag, eltrombopag, and romiplostim showed a significantly better platelet response than the placebo (OR, 36.90; 95%CI, 13.33–102.16; OR, 19.33; 95%CI, 8.42–44.40; OR, 11.92; 95%CI, 7.43–19.14; OR, 3.71; 95%CI, 1.27–10.86, respectively) whereas other arms such as RTX + rhTPO and RTX showed no significant differences compared with the placebo (OR, 1.73; 95%CI, 0.43–6.99; OR, 1.12; 95%CI, 0.48–2.61, respectively). Avatrombopag was significantly more effective than eltrombopag, romiplostim, RTX + rhTPO, and RTX with corresponding pooled ORs of 3.10 (1.01, 9.51), 9.96 (2.29, 43.29), 21.31 (3.78, 119.98) and 33.09 (8.76, 125.02), respectively (Figure 4A). No significant differences were observed between avatrombopag and lusutrombopag (OR, 1.91; 95%CI, 0.52–7.05). Lusutrombopag showed a better platelet response than romiplostim, RTX + rhTPO, and RTX with significance (OR, 5.21; 95%CI, 1.54–17.62; OR, 11.16; 95%CI, 2.16–57.62; OR, 17.34; 95%CI, 5.15–58.36, respectively). More network of comparisons can be viewed in the Supplementary Table S1. No significant differences were observed between lusutrombopag and eltrombopag (OR, 1.62; 95%CI, 0.63–4.17). The other platelet responses in each therapeutic arm are shown in Figure 3B.

**FIGURE 5:**
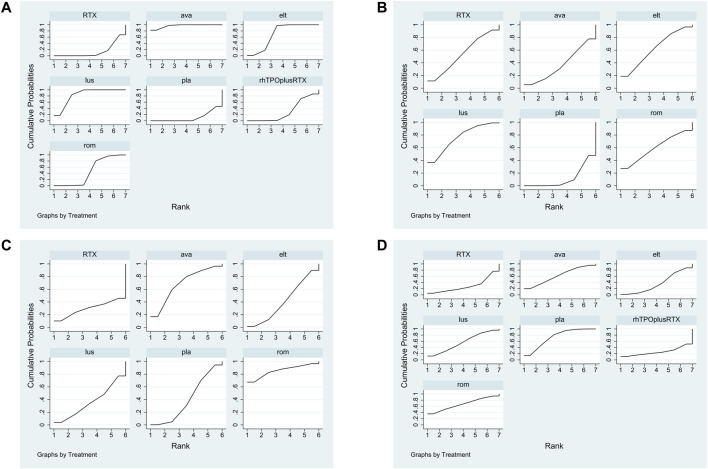
The surface under the cumulative ranking curve (SUCRA) is shown for each treatment. **(A)** Platelet response. **(B)** Any bleeding. **(C)** Composite serious adverse events. **(D)** thrombosis (ava, avatrombopag; elt, eltrombopag; rom, romiplostim; RTX, rituximab; lus, lusutrombopag).

**FIGURE 6:**
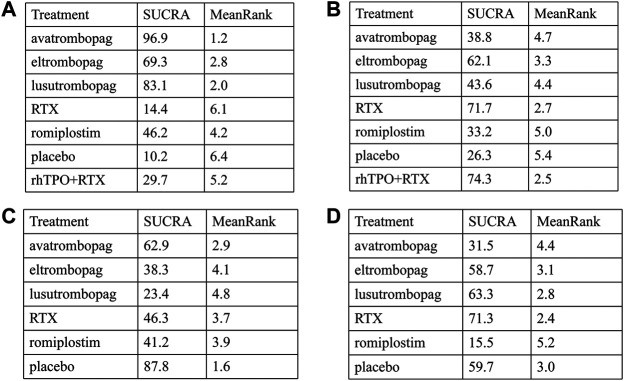
SUCRA rank of each intervention. **(A)** Platelet response. **(B)** Thrombosis. **(C)** Any bleeding. **(D)** Composite serious adverse events (ava, avatrombopag; elt, eltrombopag; rom, romiplostim; RTX, rituximab; lus, lusutrombopag).

The SUCRA was showed in Figure 5A. Avatrombopag was ranked as the best treatment for platelet response according to its SUCRA value of 96.9, followed by lusutrombopag (83.1), eltrombopag (69.3), romiplostim (46.2), rhTPO + rituximab (29.7), rituximab (14.4), and placebo (10.2) (Figure 6A). These data indicate that the patients had the highest probability of achieving PR when treated with avatrombopag. The small size of the rhTPO + rituximab arm (1 study) should be noted when drawing conclusions from these findings.

#### Bleeding

Twelve studies reported bleeding outcomes. Data from these 12 studies included five direct comparisons among the five treatments (Figure 3B). The pooled results demonstrated that TPO-RAs significantly reduced the incidence of any or severe bleeding events. All possible pairwise comparisons were made, which indicated that lusutrombopag had the lowest risk for any bleeding when compared with the placebo, followed by eltrombopag, romiplostim, rituximab, avatrombopag pooled OR of 2.22 (1.08, 4.57), 1.82 (0.87, 3.82), 1.82 (0.58, 5.73), 1.62 (0.76, 3.47), and 1.29 (0.59, 2.83), respectively (Figure 4B). More network of comparisons can be viewed in the Supplementary Table S2. However, none of the placebo and active controlled comparisons were statistically significant, except for lusutrombopag versus placebo. Lusutrombopag was ranked as the best treatment for bleeding. There was no evidence of inconsistencies or publication bias.

According to the SUCRA values (Figure 5B), lusutrombopag was ranked as the best treatment for bleeding, with a SUCRA value of 23.4, followed by eltrombopag (38.3), romiplostim (41.2), rituximab (46.3), and avatrombopag (62.9), respectively (Figure 6C). Episodes of bleeding were generally observed in patients with little or no platelet response to the therapeutic arms.

#### Incidence of Severe Adverse Events

In total, 13 studies included data regarding severe adverse events (CTCAE grade 3 or more) related to each intervention. These comprised five direct comparisons among the five treatments (Figure 3C). The criteria for severe adverse effects and adverse effects were shown in these included trials; some severe adverse events included thrombosis, acute myocardial infarction, and hypotension. All possible pairwise comparisons were made (Figure 4C), and RTX was found to have the highest risk of severe adverse events compared to the placebo, followed by lusutrombopag, eltrombopag, avatrombopag, with a pooled OR of 1.66 (0.14, 19.41), 1.10 (0.42, 2.89), 0.99 (0.62, 1.58), 0.65 (0.28, 1.55), respectively. More network of comparisons can be viewed in the Supplementary Table S4. The pooled data showed no significant differences in severe adverse events between patients receiving the five types of interventions. There was no evidence of inconsistencies. The SUCRA (Figure 5D) rankings revealed that romiplostim carries the least severe adverse events risk (15.5), whereas RTX carries the highest risk (71.3). Additionally, lusutrombopag (63.3), eltrombopag (58.7), and avatrombopag (31.5) were associated with a modest risk (Figure 6D).

## Discussion

Traditional meta-analysis only analyzed the effect of TPO-RAs with placebo or for specific thrombocytopenia (H et al.; Zhang et al., 2017). These are not comprehensive assessments of TPO-RAs. Our network meta-analysis provides unified hierarchies of evidence for ITP with five TPO-RAs in adults, thus overcoming the absence of comparative data in head-to-head trials. All TPO-RAs were found to be superior to the placebo in terms of platelet response. The magnitude of treatment effect estimates varied greatly across different TPO-RAs. Lusutrombopag and avatrombopag were ranked as the most effective agents in terms of platelet response. Both showed significant differences compared with the placebo. However, the risk of adverse events and bleeding was higher in patients treated with avatrombopag. Considering the clinical efficacy and adverse events simultaneously by clustered ranking indicated that lusutrombopag was the treatment with the best balance between high short-term efficacy with regard to platelet response, platelet count, risk of bleeding, and adverse events. Data from these studies also suggested the clinically important effects of eltrombopag and romiplostim. Rituximab appeared to have the lowest clinical efficacy and a higher risk of bleeding and thrombosis. The effects on TPO-RAs and rituximab were different according to the treatment regimens, consistent with their pharmacological effects.

Because of the higher incidence of thrombosis in patients with ITP than in the healthy population, it was recognized as a unique adverse event (Doobaree et al., 2016). However, the pathogenic mechanisms responsible for the increased thrombotic risk associated with TPO-RAs have not been identified (Kado and McCune, 2019). A recent study reported excessive increased platelet count in patients treated with TPO-RAs, and stimulated the production of young, more active platelets may be the reason for high risk of thrombosis (Rodeghiero, 2016). In fact, the overall results from our network meta-analysis indicated that no significant differences were observed between the TPO-RAs and placebo (Figure 4D). So the result of thrombosis Figures 3D, 5D, and 6C was not explained in details. Furthermore, the need for new studies to research the mechanism of thrombosis would help us better understanding and use of TPO-RAs.

For patients with persistent and chronic ITP, reducing the incidence of severe bleeding may be more important than achieving specific platelet counts. Our meta-analysis demonstrated that TPO-RAs were beneficial for patients with ITP in terms of other outcomes, but the difference was not statistically significant; further, most bleeding events were mild to moderate in severity and did not increase in frequency or severity over time. Our data suggests no significant differences in severe adverse events among the investigated agents, likely because neither of the TPO-RAs studies were sufficient to assess safety. Most adverse events appeared to be mild to moderate, and resolved either spontaneously or after medical intervention. These are the reasons why TPO-RAs may be first choice for the second-line treatment of ITP.

So far, the pathogenic mechanisms responsible for ITP are not fully researched. It is necessary to predict responses to specific treatments (Cooper and Ghanima, 2019). The recommendation of American Society of Hematology 2019 guidelines for ITP is still corticosteroids in the first-line. The second-line treatment of ITP is in favor of TPO-RA rather than rituximab (Neunert et al., 2019). This suggests that TPO-RAs have great market prospect, great potential and with high market share. However, the price of these drugs is relatively expensive, which results in a limited audience. Our study had several potential limitations. First, because of the scant primary data, the durable effects of treatments with TPO-RAs are very uncertain, which is a pivotal weakness in our understanding of these drugs. The present debate be solved only by collecting robust data for these outcomes in future trials. Second, only one study of rhTPO versus rituximab was included in our network meta-analysis, and there was no direct arm between rhTPO and other TPO-RAs. These outcomes do not allow us to properly estimate the risk-benefit ratios. Therefore, large-scale, rigorously designed, multi-center randomized clinical trials are needed to verify the efficacy and safety of rhTPO. Third, we did not control for dose in our analyses; in most of the studies included, clinicians were allowed to titrate drug doses for individual participants, which led to clinically unimportant differences in platelet outcomes.

In summary, the current study demonstrated that avatrombopag has the most beneficial effect as a second-line treatment in the short term for adults with thrombocytopenia. Recombinant human thrombopoietin may not be beneficial because of its lower efficacy and higher complications compared to other TPO-RAs. However, this was not a direct comparison. Future worldwide head-to-head RCTs including these regimens (TPO-RAs vs placebo, or direct comparison including eltrombopag, romiplostim, avatrombopag, lusutrombopag, and rhTPO) are essential, to validate our results and to determine the most suitable therapeutic strategies for persistent or chronic ITP in adults.

## Data Availability

The original contributions presented in the study are included in the article/Supplementary Material, further inquiries can be directed to the corresponding authors.
