# Supplementary material for: Comparative Efficacy and Safety of Thrombopoietin Receptor Agonists in Adults With Thrombocytopenia: A Systematic Review and Network Meta-analysis of Randomized Controlled Trial
Source: Front Pharmacol. 2021 Jul 28;12:704093. doi: 10.3389/fphar.2021.704093 (PMC8355583; doi:10.3389/fphar.2021.704093)
Supplement: Supplementary file 1 [file datasheet1.docx]

Supplementary Material

# Supplementary Table 1

**Table 1** The network of comparisons included in the network meta-analysis for platelet response

| Avatrombopag | Lusutrombopag | Eltrombopag | Romiplostim | rhTPOplusRTX | RTX | placebo |
| --- | --- | --- | --- | --- | --- | --- |
| Avatrombopag | 0.52 (0.14,1.94) | 0.32(0.11,0.99) | 0.10(0.02,0.44) | 0.05(0.01,0.26) | 0.03(0.01,0.11) | 0.03(0.01,0.08) |
| 1.91(0.52,7.05) | Lusutrombopag | 0.62(0.24,1.59) | 0.19(0.06,0.65) | 0.09(0.02,0.46) | 0.06(0.02,0.19) | 0.05(0.02,0.12) |
| 3.10(1.01,9.51) | 1.62(0.63,4.17) | Eltrombopag | 0.31(0.10,0.99) | 0.15(0.03,0.63) | 0.09(0.04,0.25) | 0.08(0.05,0.13) |
| 9.96(2.29,43.29) | 5.21(1.54,17.62) | 3.22(1.01,10.24) | Romiplostim | 0.47(0.08,2.79) | 0.30(0.07,1.22) | 0.27(0.09,0.79) |
| 21.31(3.78,119.98) | 11.16(2.16,57.62) | 6.88(1.57,30.09) | 2.14(0.36,12.77) | rhTPOplusRTX | 0.64(0.21,1.94) | 0.58(0.14,2.33) |
| 33.09(8.76,125.02) | 17.34(5.15,58.36) | 10.69(4.02,28.41) | 3.32(0.82,13.53) | 1.55(0.51,4.69) | RTX | 0.90(0.38,2.10) |
| 36.90(13.33,102.16) | 19.33(8.42,44.40) | 11.92(7.43,19.14) | 3.71(1.27,10.86) | 1.73(0.43,6.99) | 1.12(0.48,2.61) | placebo |

# Supplementary Table 2

**Table 2.** The network of comparisons included in the network meta-analysis for any bleeding events

| Lusutrombopag | Eltrombopag | Romiplostim | RTX | Avatrombopag | placebo |
| --- | --- | --- | --- | --- | --- |
| Lusutrombopag | 1.22(0.43,3.44) | 1.22(0.31,4.72) | 1.37(0.48,3.92) | 1.72(0.59,5.01) | 2.22(1.08,4.57) |
| 0.82(0.29,2.31) | Eltrombopag | 1.00(0.25,3.91) | 1.12(0.39,3.26) | 1.41(0.48,4.16) | 1.82(0.87,3.82) |
| 0.82(0.21,3.18) | 1.00(0.26,3.92) | Romiplostim | 1.13(0.28,4.46) | 1.41(0.35,5.67) | 1.82(0.58,5.73) |
| 0.73(0.26,2.08) | 0.89(0.31,2.58) | 0.89(0.22,3.52) | RTX | 1.26(0.42,3.75) | 1.62(0.76,3.47) |
| 0.58(0.20,1.69) | 0.71(0.24,2.09) | 0.71(0.18,2.84) | 0.80(0.27,2.38) | Avatrombopag | 1.29(0.59,2.83) |
| 0.45(0.22,0.93) | 0.55(0.26,1.15) | 0.55(0.17,1.73) | 0.62(0.29,1.32) | 0.78(0.35,1.70) | placebo |

# Supplementary Table 3

**Table 3.** The network of comparisons included in the network meta-analysis for severe adverse events (Grade 3 or more according to CTCAE).

| Avatrombopag | Eltrombopag | placebo | Lusutrombopag | RTX |
| --- | --- | --- | --- | --- |
| 2.12(0.27,16.65) | 3.22(0.47,22.20) | 3.24(0.50,21.09) | 3.55(0.43,29.27) | 5.39(0.25,118.38) |
| Avatrombopag | 1.52(0.57,4.06) | 1.53(0.65,3.63) | 1.68(0.46,6.14) | 2.55(0.19,34.42) |
| 0.66(0.25,1.75) | Eltrombopag | 1.01(0.63,1.60) | 1.10(0.38,3.23) | 1.67(0.14,20.39) |
| 0.65(0.28,1.55) | 0.99(0.62,1.58) | placebo | 1.10(0.42,2.89) | 1.66(0.14,19.41) |
| 0.60(0.16,2.18) | 0.91(0.31,2.65) | 0.91(0.35,2.40) | Lusutrombopag | 1.52(0.11,21.25) |
| 0.39(0.03,5.30) | 0.60(0.05,7.28) | 0.60(0.05,7.01) | 0.66(0.05,9.24) | RTX |

# Supplementary Table 4

| placebo | Romiplostim | Avatrombopag | Lusutrombopag | Eltrombopag | _RTX | _rhTPOplusRTX |
| --- | --- | --- | --- | --- | --- | --- |
| placebo | 0.98(0.09,11.08) | 1.24(0.26,5.94) | 1.44(0.38,5.49) | 2.34(0.75,7.25) | 5.09 (0.24,108.27) | 7.68 (0.09,653.37) |
| 1.02(0.09,11.64) | Romiplostim | 1.28(0.07,22.93) | 1.48(0.09,23.65) | 2.39(0.16,34.96) | 5.21 (0.10,259.08) | 7.87 (0.05,1246.11) |
| 0.80(0.17,3.83) | 0.78(0.04,14.08) | Avatrombopag | 1.16(0.15,9.04) | 1.88(0.27,12.92) | 4.09 (0.13,126.61) | 6.17 (0.06,685.00) |
| 0.69(0.18,2.65) | 0.68(0.04,10.86) | 0.86(0.11,6.76) | Lusutrombopag | 1.62(0.28,9.37) | 3.53 (0.13,99.46) | 5.33 (0.05,552.54) |
| 0.43(0.14,1.33) | 0.42 (0.03,6.10) | 0.53(0.08,3.67) | 0.62(0.11,3.56) | Eltrombopag | 2.18 (0.08,56.80) | 3.29 (0.03,322.48) |
| 0.20(0.01,4.19) | 0.19(0.00,9.53) | 0.24(0.01,7.59) | 0.28 (0.01,7.97) | 0.46 (0.02,11.98) | RTX | 1.51 (0.06,37.94) |
| 0.13(0.00,11.08) | 0.13(0.00,20.11) | 0.16 (0.00,18.01) | 0.19(0.00,19.43) | 0.30 (0.00,29.83) | 0.66 (0.03,16.64) | rhTPOplusRTX |

1. **Search Strategy**

“TPO-RA,” “Thrombopoiesis,” “ thrombopoie,” “thrombocytopenia,” “TPO,” “thrombopoietin receptor agonists, ” “avatrombopag,” “E5501 or E-5501 or E 5501,” “AKR501 or AKR 501 or AKR‐501,” “YM477 or YM 477 or YM‐477,” “AS1670542 or AS 1670542 or AS-1670542,” “lusutrombopag,” “AK338693 or AK 338693 or AK-338693,” S-888711 or S888711 OR S 888711,” “Amgen Megakaryopoiesis protein 531,” “Nplate,” “amg531 or amg 531 or amg‐531,” “romiplostim,” “romiplastin,” “promacta,” “sb497115 or sb 497115 or sb‐497115,” “revolade,” “eltrombopag,” “mabthera,” “recombinant human thrombopoietin,” “RCT,” “randomized controlled trials,” “rhTPO,” “TPIAO,” “FAB59 or FAB 59 or FAB-59.”
